# Handgrip strength is associated with cognitive function in patients with head injury with loss of consciousness: results from the NHANES 2011–2014

**DOI:** 10.3389/fneur.2025.1572650

**Published:** 2025-07-03

**Authors:** Bilian Guo, Weihui He, Ni Zeng, Xiang Xu, Zhipeng Yan

**Affiliations:** ^1^Department of Rehabilitation Medicine, First Affiliated Hospital of Fujian Medical University, Fuzhou, China; ^2^Department of Rehabilitation Medicine, National Regional Medical Center, Binhai Campus of the First Affiliated Hospital, Fujian Medical University, Fuzhou, China; ^3^Department of Dermatology, First Affiliated Hospital of Fujian Medical University, Fuzhou, China; ^4^Department of Rehabilitation Medicine, Affliated Hospital of Guizhou Medical University, Guiyang, China

**Keywords:** head injury with loss of consciousness, handgrip strength, cognitive function, gender, NHANES

## Abstract

**Background:**

We evaluated the relationship between handgrip strength (HGS) and cognitive function in patients with head injury with loss of consciousness (HIC) using National Health and Nutrition Examination Survey (NHANES) data.

**Methods:**

Utilizing the 2011–2014 NHANES database, we screened participants who completed the HIC questionnaire and extracted relevant variables. Key variable was the sum of maximum bilateral HGS. Cognitive function encompassed global cognitive function, Immediate Recall Test (IRT), Delayed Recall Test (DRT), Animal Fluency Test (AFT), and Digit Symbol Substitution Test (DSST). Weighted multivariate linear regression analyzed the relationship. Gender-stratified analyses explored differences. Restricted cubic spline models elucidated the dose–response curves of HGS on cognitive function.

**Results:**

Among 283 American HIC patients, HGS significantly correlated positively with global cognitive function, IRT, DRT, and DSST. Gender-stratified analysis showed that HGS enhancement significantly improved DRT and DSST scores in males, while females primarily benefited in Global cognitive function and AFT. Restricted cubic spline analysis confirmed a consistent linear dose–response relationship between HGS and cognitive function indicators, valid in both genders.

**Conclusion:**

Our study reveals a positive correlation between HGS and cognitive function in HIC patients, with gender differences, offering a novel perspective for cognitive status assessment. Future large-scale, multidimensional studies are needed to deepen understanding of the complex HGS-cognitive function relationship.

## Introduction

Head injury stands as a significant contributor to disability and mortality worldwide, exerting profound impacts. It represents a highly heterogeneous disease spectrum with a wide range of injury severity, encompassing conditions from minor scalp abrasions to severe traumatic brain injuries. Among these, loss of consciousness (LOC) is frequently regarded as a cardinal indicator of severe head trauma, and its occurrence is typically associated with functional impairment of neural circuits responsible for maintaining conscious awareness (1). In the United States, a staggering 16% of adults aged 40 and above report a history of head trauma accompanied by loss of consciousness (2, 3). Compared to head injuries without LOC, those accompanied by LOC are often closely associated with more severe degrees of brain injury. Current research indicates that such injuries are not only significantly linked to poor prognostic outcome but may also lead to long-term risks of cognitive decline (4, 5), encompassing memory impairment, reduced information processing speed, and compromised executive function, with the extreme possibility of progressing into dementia.

In recent years, handgrip strength (HGS), a simple and easily measurable indicator of muscular strength, has emerged as a novel research value in assessing health status, particularly in exploring its correlation with cognitive function (6–8). While existing studies have yielded divergent findings regarding the relationship between grip strength and cognitive decline as well as dementia risk, compelling evidence points to a trend: lower baseline grip strength levels often predict faster rates of cognitive decline and a higher incidence of dementia (9–11). For instance, the National Institute on Aging’s Longitudinal Study of Aging (NILS-LSA) in the United States, utilizing standardized tests such as the Mini-Mental State Examination (MMSE) and the Digit Symbol Substitution Test (DSST), has revealed the predictive role of low grip strength in cognitive decline over a 10-year period (10). Nevertheless, some studies conducted in broader populations have failed to directly establish a definitive link between grip strength and specific cognitive impairments (12, 13), underscoring the multifaceted and complex nature of cognitive function.

Given the inconsistency in the aforementioned findings and the multidimensional nature of cognitive function assessment, this study innovatively concentrates its attention on the unique cohort of patients experiencing head injury with loss of consciousness (HIC). Utilizing the extensive data resources from the National Health and Nutrition Examination Survey (NHANES) conducted between 2011 and 2014, and by integrating various cognitive assessment tools, this study aims to comprehensively and systematically analyze the potential relationship between handgrip strength and cognitive performance among patients with HIC.

## Materials and methods

### Study population

**Figure 1:**
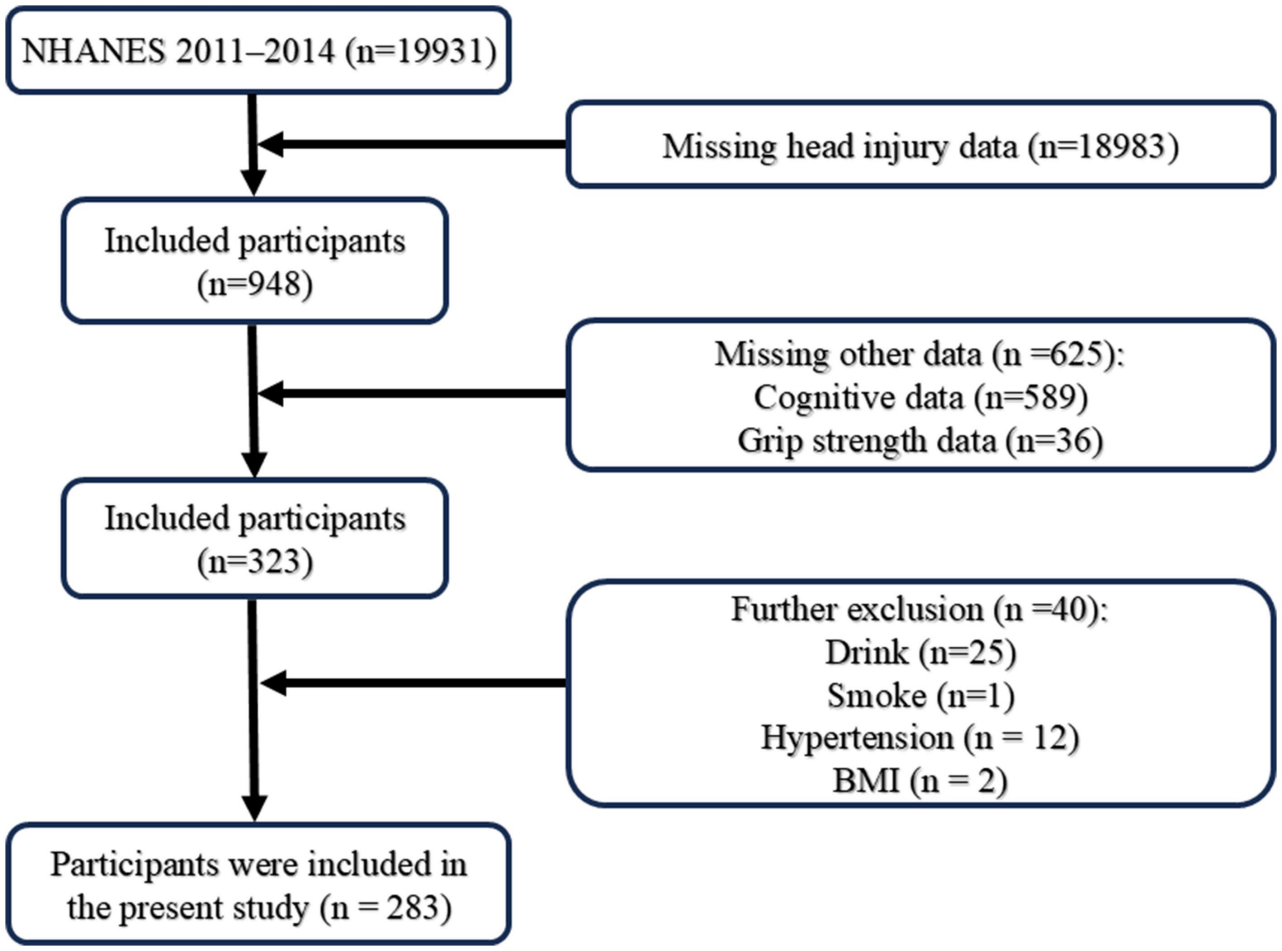
Diagrammatic representation of the participant selection process.

The study has received approval from the National Center for Health Statistics research ethics review board, and all participants have signed informed consent forms. NHANES is a cross-sectional study administered by the Centers for Disease Control and Prevention (CDC) in the United States, conducted every 2 years since 1999 to assess the health and nutritional status of adults and children in the country. Employing a multi-stage probability sampling method, NHANES ensures the representativeness of its sample, with participants required to complete household interviews, physical examinations, and biological sample collections. This study utilized data from the 2011–2012 and 2013–2014 survey cycles to evaluate head injuries.^1^ The question regarding head injuries with loss of consciousness was part of a taste and smell questionnaire administered to participants, which read, “Have you ever had a loss of consciousness because of a head injury?” Participants who responded “yes” to this question were classified as having experienced a HIC. Among the 19,931 participants who completed the questionnaire, we excluded those with missing information on “head injury (*n* = 18,983), cognitive data (*n* = 589), HGS (*n* = 36)” and sociodemographic data, including alcohol consumption (*n* = 25), smoking (*n* = 1), hypertension (*n* = 12), and body mass index (BMI) (*n* = 2). Ultimately, 283 participants were included in the analysis, and the screening process is shown in Figure 1.

### HGS assessment

In accordance with the standards set forth by the Medical Research Institute, the NHANES study employed the T. K. K.5401 digital hand dynamometer manufactured by Takei Scientific Instruments (Niigata, Japan) to precisely evaluate individual grip strength in kilograms (kg). Trained examiners guided the participants and adjusted the grip handles according to their hand sizes. Following an initial trial grip, participants were instructed to squeeze the dynamometer with maximal effort while exhaling. This process was repeated three times for each hand, alternating between hands, with a 60-s rest interval between each measurement. Unless physically constrained, all tests were conducted in a standing position to mimic natural conditions. Participants who were unable to complete the test due to reasons such as missing arms/hands/thumbs, scheduling conflicts, refusal, or health issues were considered ineligible. For a comprehensive understanding of the testing procedures and operational norms, please refer to the NHANES Muscle Strength Procedures Manual. Consistent with prior research utilizing similar methodologies, the sum of maximum grip strength from both hands was adopted as the key exposure variable in this study (14).

### Cognitive assessment

The NHANES 2011–2014 cognitive assessments comprehensively evaluated participants’ cognitive faculties, encompassing domains such as immediate memory, delayed memory, categorical verbal fluency, and processing speed. These evaluations encompassed standardized tests from the Consortium to Establish a Registry for Alzheimer’s Disease (CERAD), specifically the Word List Learning Test (also known as the Immediate Recall Test, IRT), Delayed Recall Test (DRT) (15), Animal Fluency Test (AFT) (16), and the Digit Symbol Substitution Test (DSST) (17). Elevated scores across all tests are indicative of superior cognitive function.

Within the IRT, participants underwent a series of three consecutive learning trials, wherein they were presented with ten unrelated words per trial and tasked with recalling as many as possible. The order of the words varied across trials, and the IRT total score, a cumulative measure of performance across the three trials, ranged up to 30, serving as a robust indicator of immediate memory capacity.

The DRT, on the other hand, evaluated delayed memory by requiring participants to retrieve the words learned during the IRT. Scoring for this test was based solely on performance during a single recall trial.

The AFT, a metric of executive function, specifically assessed categorical verbal fluency by prompting participants to enumerate as many animal names as possible within a one-minute time frame. Each correctly named animal contributed to the participant’s score.

Furthermore, the DSST, a component of the Wechsler Adult Intelligence Scale (WAIS III), was administered to assess processing speed, sustained attention, and working memory. During this test, participants utilized a paper form to accurately match symbols with corresponding digits within 133 boxes, within a two-minute timeframe. The score was determined by the number of correct matches achieved.

To ensure comparability across assessments, which inherently possessed varying maximum scores, all cognitive scores were standardized into z-scores. This process involved subtracting the standard mean from each participant’s raw score and dividing the result by the standard deviation. Ultimately, a composite score, which serves as a holistic representation of overall cognitive function, was derived by calculating the average of the standardized scores obtained from the four cognitive tests.

### Covariates assessments

In the ultimate analysis, we incorporated various covariates to adjust our findings. The demographic data collected through questionnaires encompassed respondents’ age (treated as a continuous variable), gender (male or female), race (including non-Hispanic White, non-Hispanic Black, Mexican American, and Other), and educational attainment (stratified into three levels: less than high school, high school or equivalent, and college or above). Information on alcohol consumption was categorized as Never drink, Former drink, and Current drink, while smoking status was divided into Never smoker, Former smoker, and Current smoker, based on whether the participant had smoked at least 100 cigarettes in their lifetime and their current smoking habits. BMI was calculated as weight in kilograms divided by the square of height in meters. Diabetes was diagnosed based on self-reported physician diagnosis, current use of antidiabetic medications, or a glycated hemoglobin (HbA1c) level ≥7%. Hypertension was identified according to self-reported physician diagnosis, current use of antihypertensive medications, or blood pressure readings of systolic ≥140 mmHg or diastolic ≥90 mmHg. Additionally, self-reported history of cardiovascular disease (specifically, congestive heart failure, coronary heart disease, angina/angina pectoris, or heart attack) was included. Finally, depression was defined by the 9-item Patient Health Questionnaire (PHQ-9) score, with a threshold of 10 or above indicating the presence of depression (18, 19).

### Statistical analysis

This study meticulously delineates the characteristics of the study population, presenting continuous variables as mean ± standard deviation and categorical variables as frequencies. Chi-square tests and independent samples t-tests were utilized to evaluate gender differences in categorical and continuous variables, respectively. To ensure the representativeness of the analysis, the “full-sample 2-year MEC examination weight” was applied to the 2011–2014 data. A stepwise approach to constructing multivariate weighted linear regression models was employed to investigate the association between grip strength and cognitive function among patients with HIC, along with its gender-specific manifestations: Model 1 adjusted for fundamental demographic characteristics including age, sex, race/ethnicity, and educational attainment in the regression analysis; Model 2 extended Model 1 by incorporating lifestyle-related variables such as drinking habits, smoking status, and BMI; Model 3 further expanded upon Model 2 by including health indicators like depression, diabetes, hypertension, and cardiovascular disease. Finally, Restricted Cubic Splines (RCS) were applied to analyze the linear relationship between grip strength and cognition in HIC patients, with gender-stratified analyses conducted to explore potential differences. All analyses were performed using R software (version 4.2.2), with statistical significance set at *p* < 0.05.

## Results

### Baseline characteristics

The baseline characteristics of HIC patients from NHANES 2011–2014 are presented in Table 1. Our final analysis encompassed 283 HIC patients aged 60 and above, comprising 170 males and 113 females. The mean age of the patients was 68.55 ± 6.75 years. The majority of the patients were non-Hispanic whites, accounting for 178 individuals (105 males and 73 females). Furthermore, a substantial proportion of the patients had attained an educational level beyond high school, with 183 patients (109 males and 74 females) meeting this criterion. In terms of alcohol consumption, 191 patients reported recent alcohol intake, with 119 males and 72 females. Regarding smoking status, 46 patients were current smokers, including 32 males and 14 females. Male patients exhibited an average BMI of 28.38 ± 5.26, while females had a BMI of 29.94 ± 7.34. Regarding comorbidities, we observed that 43 patients suffered from depression, 70 had diabetes, 188 were hypertensive, and 66 patients had cardiovascular disease. Notably, males demonstrated significantly stronger HGS than females, with mean values of 76.60 ± 15.12 kg and 48.38 ± 10.34 kg, respectively. Additionally, significant differences were observed between male and female HIC patients in terms of alcohol consumption, smoking status, depression, and HGS. Beyond these differences, our analysis also revealed significant variations in the scores of IRT, DRT, DSST, and global cognitive function between male and female HIC patient cohorts. These findings provide crucial insights into the gender-specific impacts on cognitive function and physiological indicators among HIC patients.

**Table 1:** Baseline characteristic of the study population.

**Characteristics**	**Overall (unweighted *n* = 283)**	**Male (unweighted *n* = 170)**	**Female (unweighted *n* = 113)**	***p* Value**
Age (yeas)	68.55 ± 6.75	68.61 ± 6.68	68.48 ± 6.88	0.876
Race/ethnicity (*N*),%				0.486
Non-Hispanic White	178 (62.9)	105 (61.8)	73 (64.6)	
Non-Hispanic Black	35 (12.4)	25 (14.7)	10 (8.8)	
Mexican American	19 (6.7)	10 (5.9)	9 (8.0)	
Other	51 (18.0)	30 (17.6)	21 (18.6)	
Education (*N*), %				0.42
Less than high school	25 (8.8)	18 (10.6)	7 (6.2)	
High school or equivalent	75 (26.5)	43 (25.3)	32 (28.3)	
Greater than high school	183 (64.7)	109 (64.1)	74 (65.5)	
Drink (*N*), %				**<0.001**
Never drink	30 (10.6)	8 (4.7)	22 (19.5)	
Former drink	62 (21.9)	43 (25.3)	19 (16.8)	
Current drink	191 (67.5)	119 (70.0)	72 (63.7)	
Smoke (*N*), %				**<0.001**
Never smoker	102 (36)	43 (25.3)	59 (52.2)	
Former smoker	135 (47.7)	95 (55.9)	40 (35.4)	
Current smoker	46 (16.3)	32 (18.8)	14 (12.4)	
BMI	29.00 ± 6.21	28.38 ± 5.26	29.94 ± 7.34	0.053
Depression (*N*), %				**0.049**
Yes	43 (15.2)	20 (11.8)	23 (20.4)	
No	240 (84.8)	150 (88.2)	90 (79.6)	
Diabetes (*N*), %				0.564
Yes	70 (24.7)	40 (83.5)	30 (26.5)	
No	213 (75.3)	130 (76.5)	83 (73.5)	
Hypertension (*N*), %				0.205
Yes	188 (66.4)	108 (63.5)	80 (70.8)	
No	95 (33.6)	62 (36.5)	33 (29.2)	
History of cardiovascular disease (*N*), %				0.068
Yes	66 (23.3)	46 (27.1)	20 (17.7)	
No	217 (76.7)	124 (72.9)	93 (82.3)	
Global cognitive function	0.0003 ± 0.7888	−0.1118 ± 0.8007	0.1690 ± 0.7413	**0.003**
IRT	19.33 ± 4.60	18.72 ± 4.54	20.25 ± 4.56	**0.006**
DRT	6.33 ± 2.25	5.95 ± 2.31	6.9 ± 2.02	**<0.001**
AFT	18.06 ± 5.72	18.24 ± 5.89	17.8 ± 5.46	0.528
DSST	49.48 ± 17.00	46.45 ± 16.85	54.04 ± 16.27	**<0.001**
Handgrip strength (kg)	65.33 ± 19.27	76.60 ± 15.12	48.38 ± 10.34	**<0.001**

BMI, Body Mass Index; IRT, Immediate Recall Test; DRT, Delayed Recall Test; AFT, Animal Fluency Test; DSST, Digital Symbol Substitution Test. Significant values (*p* < 0.05) are in bold.

### Association between HGS and cognitive function

Table 2 delves into the potential associations between HGS and an array of cognitive test performances among patients with HIC using a multivariate linear regression model. Within the specific analytical framework of Model 3, the findings underscored a significant positive correlation between higher HGS levels and better performance on global cognitive function, IRT, DRT, and DSST, excluding the factor of AFT. This discovery emphasized the potential link between stronger HGS and superior cognitive outcomes. The linear regression coefficients and their corresponding 95% confidence intervals (95% CI) were as follows: 0.009 (0.003, 0.016) for global cognitive score, 0.045 (0.004, 0.087) for IRT score, 0.032 (0.012, 0.053) for DRT score, and 0.191 (0.067, 0.315) for attention score as assessed by DSST.

**Table 2:** Multiple linear regression analysis of handgrip strength and cognitive score.

Cognitive score	Overall		Male		Female	
	*β* (95%CI)	*p*	*β* (95%CI)	*p*	*β* (95%CI)	*p*
Global cognitive function						
Model 1^a^	**0.007 (0.001, 0.014)**	**0.021**	0.004 (−0.004, 0.011)	0.336	**0.016 (0.003, 0.029)**	**0.018**
Model 2^b^	**0.009 (0.003, 0.016)**	**0.005**	0.006 (−0.002, 0.013)	0.143	**0.017 (0.004, 0.031)**	**0.012**
Model **3**^**c**^	**0.009 (0.003, 0.016)**	**0.005**	0.007 (−0.001, 0.015)	0.072	**0.014 (0.000, 0.028)**	**0.043**
IRT						
Model 1^a^	0.031 (−0.008, 0.071)	0.119	0.021 (−0.025, 0.066)	0.371	0.064 (−0.022, 0.149)	0.141
Model 2^b^	**0.041 (0.000, 0.081)**	**0.049**	0.033 (−0.015, 0.081)	0.175	0.071 (−0.018, 0.159)	0.116
Model **3**^**c**^	**0.045 (0.004, 0.087)**	**0.032**	0.044 (−0.004, 0.092)	0.073	0.065 (−0.027, 0.158)	0.165
DRT						
Model 1^a^	**0.028 (0.009, 0.048)**	**0.005**	**0.028 (0.005, 0.052)**	**0.020**	0.018 (−0.020, 0.057)	0.342
Model 2^b^	**0.032 (0.012, 0.052)**	**0.002**	**0.033 (0.008, 0.058)**	**0.010**	0.023 (−0.016, 0.063)	0.237
Model **3**^**c**^	**0.032 (0.012, 0.053)**	**0.002**	**0.034 (0.009, 0.060)**	**0.008**	0.021 (−0.020, 0.061)	0.309
AFT						
Model 1^a^	0.002 (−0.053, 0.057)	0.934	−0.044 (−0.111, 0.024)	0.201	**0.125 (0.021, 0.228)**	**0.019**
Model 2^b^	0.012 (−0.043, 0.067)	0.668	−0.041 (−0.106, 0.024)	0.217	**0.134 (0.028, 0.239)**	**0.014**
Model **3**^**c**^	0.010 (−0.046, 0.065)	0.737	−0.034 (−0.101, 0.033)	0.315	**0.110 (0.001, 0.220)**	**0.048**
DSST						
Model 1^a^	**0.171 (0.045, 0.297)**	**0.008**	0.087 (−0.045, 0.219)	0.197	**0.330 (0.035, 0.625)**	**0.029**
Model 2^b^	**0.205 (0.079, 0.331)**	**0.002**	**0.136 (0.008, 0.264)**	**0.037**	**0.342 (0.033, 0.651)**	**0.031**
Model **3**^**c**^	**0.191 (0.067, 0.315)**	**0.003**	**0.152 (0.026, 0.278)**	**0.018**	0.240 (−0.066, 0.546)	0.123

^a^Adjusted for age, sex, race/ethnicity, educational attainment.

^b^Continues to adjust drinking, smoking, and BMI.

^c^Continues to adjust depression, diabetes, hypertension, and cardiovascular disease.

IRT, Immediate Recall Test; DRT, Delayed Recall Test; AFT, Animal Fluency Test; DSST, Digital Symbol Substitution Test. Significant values (*p* < 0.05) are in bold.

A deeper dive into Model 3 revealed gender-specific patterns. Among male HIC patients, an increase in HGS significantly correlates with improved DRT and DSST scores. Specifically, a 1-kg increment in HGS among male patients was projected to lead to a 0.034 increase in DRT score (95% CI: 0.009, 0.060) and a 0.152 increase in DSST score (95% CI: 0.026, 0.278). Conversely, in female HIC patients, an improvement in HGS significantly associated with enhanced global cognitive function and AFT scores. For every 1-kg increment in HGS among female patients, global cognitive function score was anticipated to increase by 0.014 (95% CI: 0.000, 0.028), while AFT score was projected to rise by 0.110 (95% CI: 0.001, 0.220).

### Dose–response association between HGS and cognitive

**Figure 2:**
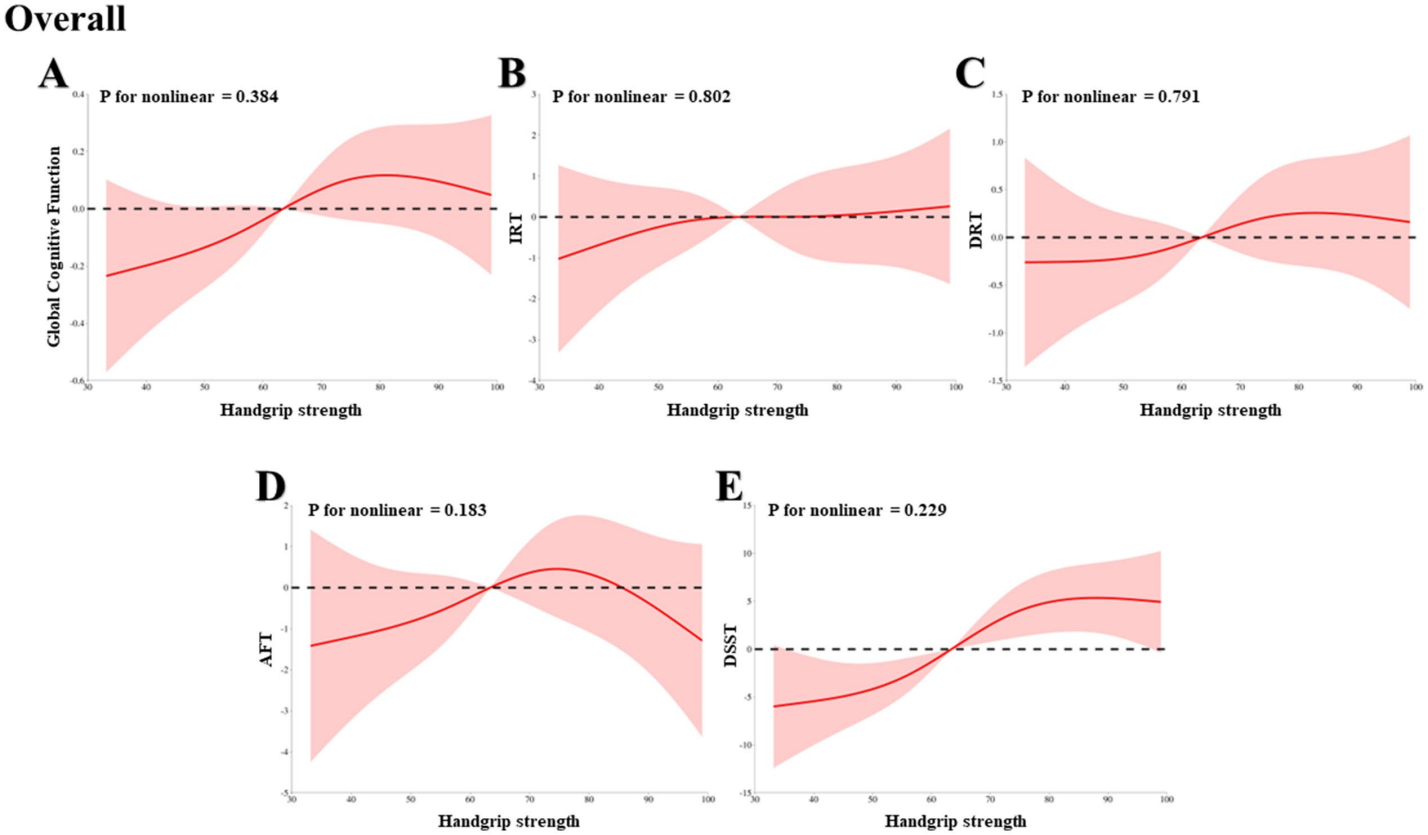
The dose–response association between HGS and cognitive in overall HIC patients based on RCS analysis. **(A)** Represents global cognitive function. **(B)** Represents IRT. **(C)** Represents DRT. **(D)** Represents AFT. **(E)** Represents DSST.

**Figure 3:**
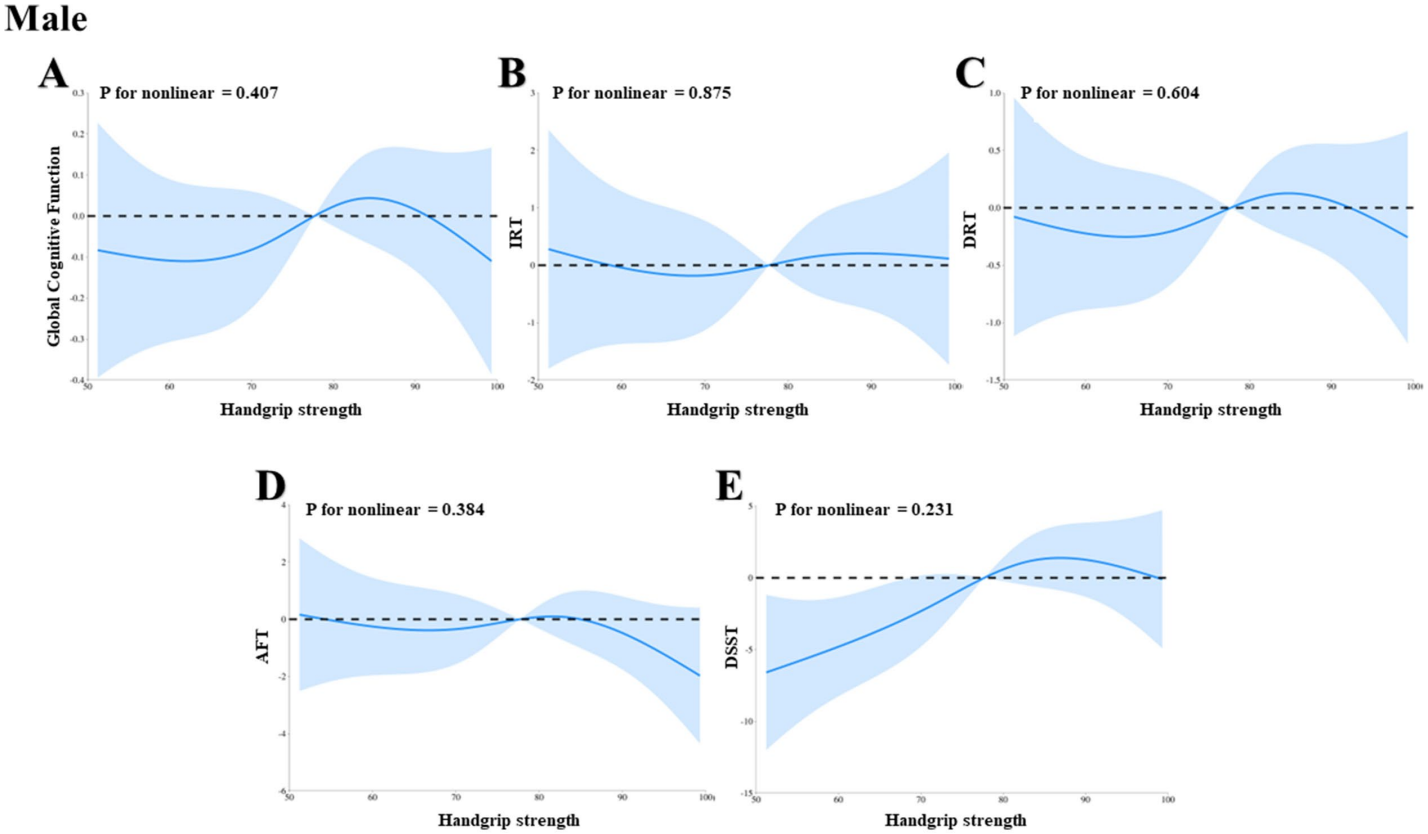
The dose–response association between HGS and cognitive in male HIC patients based on RCS analysis. **(A)** Represents global cognitive function. **(B)** Represents IRT. **(C)** Represents DRT. **(D)** Represents AFT. **(E)** Represents DSST.

**Figure 4:**
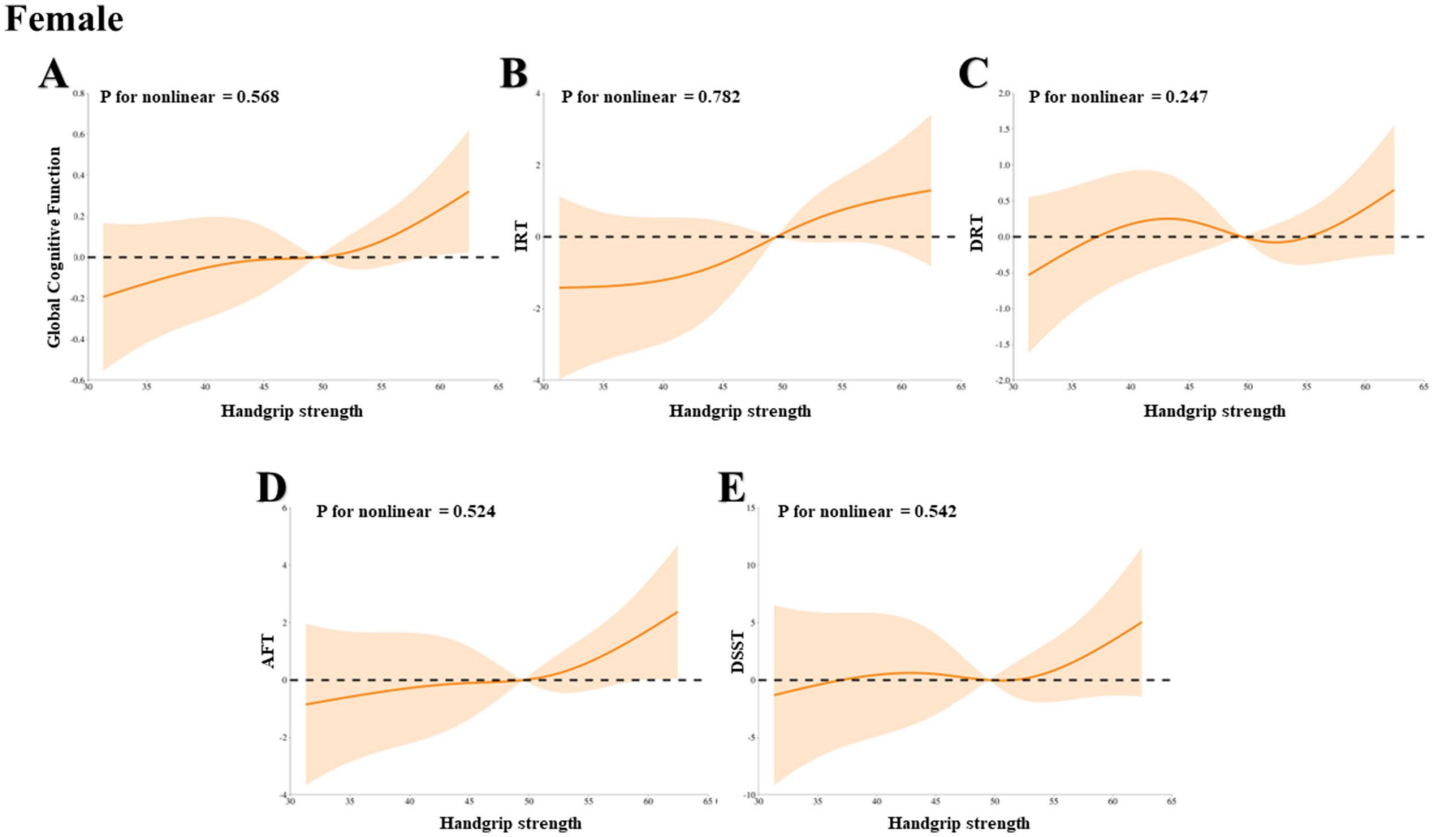
The dose–response association between HGS and cognitive in female HIC patients based on RCS analysis. **(A)** Represents global cognitive function. **(B)** Represents IRT. **(C)** Represents DRT. **(D)** Represents AFT. **(E)** Represents DSST.

Through a RCS analysis based on weighted multivariable linear regression, adjusted for potential confounders, we systematically evaluated the relationship between HGS and a range of cognitive function indices in HIC patients, including global cognitive function, IRT, DRT, AFT, and DSST. Across all HIC patients, we observed significant linear associations between HGS and all aforementioned cognitive function indices, as evidenced by the Pnonlinearity values of 0.384, 0.802, 0.791, 0.183, and 0.229, respectively (Figures 2A–E). Further stratified analyses revealed that the linear relationship between HGS and global cognitive function, IRT, DRT, AFT, and DSST persisted in both male and female HIC patient subgroups, with all *p*-values exceeding 0.05, indicating the absence of significant nonlinearity (Figures 3A–E, 4A–E, respectively).

## Discussion

In this study, we conducted an in-depth analysis of data from 283 individuals within the American HIC cohort, with the primary objective of elucidating the potential associations between HGS and cognitive impairment in these patients. Employing a multivariate linear regression model and comprehensively controlling for potential confounding variables, our findings revealed a significant positive correlation between HGS and global cognitive function, IRT, DRT, as well as DSST performance. Further gender-stratified analyses indicated that among males, an increase in HGS significantly enhanced scores on the DRT and DSST, whereas for females, the primary benefits were observed in global cognitive function and AFT evaluations. Additionally, through the application of restricted cubic spline analysis, we confirmed a consistent linear dose–response relationship between HGS and these various cognitive function indices, a trend validated in both male and female patients.

A prevalent health issue in the United States and globally, HIC exhibits a strong link with cognitive dysfunction (20–22). Although the specific mechanisms underlying how HIC leads to cognitive decline and dementia remain unclear, numerous studies have pointed to a significant increase in the incidence of cognitive impairment and dementia among individuals with a history of head injury (23, 24). This impairment in cognitive function is manifested primarily by slowed processing speed, degraded executive function, and diminished memory, significantly impacting individuals’ quality of life (5, 24). To our knowledge, the present study is the first to focus on exploring the relationship between HGS and cognition in HIC patients. HGS, a simple and non-invasive assessment of physical health, has been widely used in various research and clinical settings and is considered an important biomarker of health status in the elderly (25). Kuo et al.’s research revealed a positive association between increased absolute HGS and reduced risk of all-cause dementia and Alzheimer’s disease, providing robust support for HGS as a potential indicator of cognitive health (26). Furthermore, large-scale studies based on the UK Biobank not only confirmed the positive correlation between HGS and cognitive function but also delved into the extensive connections between HGS and increased gray matter volume, particularly in subcortical regions and the temporal cortex, which are closely related to cognitive function (27). This discovery offers a novel perspective on how HGS may influence brain structure and promote cognitive health. Additionally, Peng et al.’s study emphasized the predictive role of severe sarcopenia in cognitive impairment among the elderly, particularly the independent risk factor of poor HGS for cognitive impairment in men (28). Other cross-sectional studies have also shown an association between high HGS and lower risk of cognitive impairment, encompassing multiple cognitive domains such as memory, language, and attention (14, 29). These findings align with our current results, indicating a significant positive correlation between HGS and immediate memory, delayed memory, and attention in the US population with HIC, suggesting that HGS may serve as a useful tool for predicting cognitive recovery potential in HIC patients and that enhancing HGS may improve their cognitive levels. Notably, some studies have failed to find a significant association between HGS and cognitive function (30, 31), which may be attributed to differences in study design, sample characteristics, and assessment tools. Therefore, future research, both cross-sectional and longitudinal, in related fields is necessary to confirm the complex relationship between HGS and cognitive function.

Gender differences have consistently been a non-negligible aspect in studies exploring the relationship between HGS and cognitive function in HIC. While some studies suggesting that decreased HGS adversely impacts cognitive function similarly in both males and females, manifested as memory decline and increased dementia risk (9, 32), the specific role of gender exhibits complex and varied characteristic. Chen et al.’s research sheds light on one aspect of gender difference, revealing a synergistic effect between abdominal obesity and HGS on cognitive impairment risk in males, whereas this synergy is not significant in females, further evidencing gender disparities in cognitive health pathways (33). Moreover, independent studies on hypertensive populations and elderly cancer survivors have also uncovered gender differences in the impact of HGS on cognitive function (34, 35), which align with our findings in the US HIC population. Specifically, we observed that in males, enhanced HGS is intimately associated with significant improvements in DRT and DSST scores, primarily reflecting restored memory retention and attention function. Conversely, in females, there was a dual enhancement in global cognitive function and AFT scores, suggesting that females may be more sensitive to changes in HGS with respect to global cognitive processing and semantic fluency. Consequently, these findings collectively emphasize the importance of considering gender differences when designing rehabilitation programs for patients with HIC-related cognitive impairment, to ensure more precise and personalized treatment strategies for different genders, thereby maximizing their cognitive recovery.

### Strengths and limitations

This study also has several limitations. Firstly, given that many variables in the NHANES dataset, including head injury history, are based on self-reported information from respondents, there is a clear potential for recall bias and misclassification. Secondly, the NHANES dataset lacks information on the precise date or timing of head injury. This absence of temporal data constrains our capacity to examine how the duration elapsed since the head injury may affect the outcomes under investigation. Thirdly, despite our efforts to adjust for multiple potential confounding factors, it is still difficult to completely eliminate the interference of residual confounding effects. Moreover, due to the limited number of individuals with HIC in the sample, we were unable to conduct a stratified analysis of different levels of HGS, which to some extent limits the granularity of the study’s conclusions. Finally, as a cross-sectional study, its nature inherently prevents us from directly establishing a causal relationship between HGS and cognitive impairment in individuals with HIC; we can only confirm a statistical correlation between the two. Therefore, future research should include larger-scale, both cross-sectional and longitudinal studies, to further elucidate the complex relationship between HGS and cognitive function in individuals with HIC.

## Conclusion

The results of this study indicate that higher HGS in patients with HIC is positively correlated with their short-term memory, long-term memory, and attention cognitive functions, with potential gender-specific associations. Further analysis confirms a stable linear dose–response relationship between HGS and various dimensions of cognitive function. This finding suggests HGS as a novel perspective for assessing cognitive status in HIC patients. However, to comprehensively unravel the complex relationship between the two, large-scale, multi-dimensional cross-sectional and longitudinal studies are urgently needed in the future to deepen our understanding and optimize intervention strategies.

## Data Availability

The original contributions presented in the study are included in the article/supplementary material, further inquiries can be directed to the corresponding authors.

## Footnotes

^1^ https://www.cdc.gov/nchs/nhanes/index.htm
